# A Rapid, Strong, and Convergent Genetic Response to Urban Habitat Fragmentation in Four Divergent and Widespread Vertebrates

**DOI:** 10.1371/journal.pone.0012767

**Published:** 2010-09-16

**Authors:** Kathleen Semple Delaney, Seth P. D. Riley, Robert N. Fisher

**Affiliations:** 1 Department of Ecology and Evolutionary Biology, University of California Los Angeles, Los Angeles, California, United States of America; 2 Santa Monica Mountains National Recreation Area, National Park Service, Thousand Oaks, California, United States of America; 3 San Diego Field Station, U.S. Geological Survey, San Diego, California, United States of America; Smithsonian Institution National Zoological Park, United States of America

## Abstract

**Background:**

Urbanization is a major cause of habitat fragmentation worldwide. Ecological and conservation theory predicts many potential impacts of habitat fragmentation on natural populations, including genetic impacts. Habitat fragmentation by urbanization causes populations of animals and plants to be isolated in patches of suitable habitat that are surrounded by non-native vegetation or severely altered vegetation, asphalt, concrete, and human structures. This can lead to genetic divergence between patches and in turn to decreased genetic diversity within patches through genetic drift and inbreeding.

**Methodology/Principal Findings:**

We examined population genetic patterns using microsatellites in four common vertebrate species, three lizards and one bird, in highly fragmented urban southern California. Despite significant phylogenetic, ecological, and mobility differences between these species, all four showed similar and significant reductions in gene flow over relatively short geographic and temporal scales. For all four species, the greatest genetic divergence was found where development was oldest and most intensive. All four animals also showed significant reduction in gene flow associated with intervening roads and freeways, the degree of patch isolation, and the time since isolation.

**Conclusions/Significance:**

Despite wide acceptance of the idea in principle, evidence of significant population genetic changes associated with fragmentation at small spatial and temporal scales has been rare, even in smaller terrestrial vertebrates, and especially for birds. Given the striking pattern of similar and rapid effects across four common and widespread species, including a volant bird, intense urbanization may represent the most severe form of fragmentation, with minimal effective movement through the urban matrix.

## Introduction

Habitat loss and the resulting fragmentation can have many impacts on wildlife populations. However, the effects of fragmentation may vary based on many factors including the size, configuration, and age of habitat patches, the vagility of the species in question, and the characteristics of the matrix between patches. Urban development may represent a particularly intense form of fragmentation for many animals. Species that are particularly sensitive to urban development may be quickly lost from urban areas [1], [2], [3]. For species that remain widely distributed across fragmented landscapes, connectivity and gene flow between populations may be reduced, leading to longer-term problems such as inbreeding, loss of genetic diversity, and even local extinction [4], [5], [6], [7]. If local extinction occurs, then more isolated patches will be harder to re-colonize [4]. In addition, the loss of genetic diversity within isolated patches can lead to a decrease in a species' ability to adapt to environmental change [8], [9].

An increasing number of studies of the genetic effects of fragmentation have occurred in the past decade or so, although 30–40% of these have not shown significant effects and many are in non-urban landscapes such as fragmented forests [10]. Urbanization is a common cause of fragmentation, and conservation efforts point to the extreme land use changes associated with urbanization as one of the largest threats to biodiversity [11]. However, to date, fine-scale (within 5–10 km) genetic effects of urban fragmentation have been documented for few species [12], [13], [14], [15], [16], [17], and many studies find little effect [18], [19], [20]. Moreover, studies of the genetic effects of fragmentation are overwhelmingly on a single species, and we know of no studies where genetic patterns were compared in the same urban landscape for species from different broad taxa, such as reptiles (Class *Reptilia*) and birds (Class *Aves*), and with radically different means of locomotion, such as flying and crawling.

We investigated the genetic effects of urban fragmentation on three lizards, the side-blotched lizard (*Uta stansburiana*), western skink (*Plestiodon skiltonianus*) and western fence lizard (*Sceloporus occidentalis*), and one bird, the wrentit (*Chamaea fasciata*) in Santa Monica Mountains National Recreation Area (SMMNRA), a national park near Los Angeles. The three lizard species have widespread distributions in California [21], are small in size, are still relatively common and widespread in natural habitat throughout the area [22], and have low dispersal capabilities [23], [24], [25], [26], [27]. Side-blotched lizards and fence lizards are both in the family *Iguanidae*, but side-blotched lizards are considerably smaller and prefer more open habitat. Western skinks are in a distantly-related different family (*Scincidae*) and locally prefer grassland habitat, although all three species are broadly sympatric in the region.

Wrentits are small birds (approximately 15 g) with a distribution that is limited to the west coast of North America and follows the scrub and chaparral habitat that they prefer[28]. Wrentits are monogamous, hold small (1–2.5 acres), year-round multi-purpose territories [28], and have short dispersal distances [29]. Wrentits are obviously very different phylogenetically and ecologically from the lizards and also have the ability to fly, which could potentially increase their movement across the landscape. A bird isolated in a habitat fragment could presumably simply fly over urban areas to disperse to other suitable habitats, thereby preventing genetic divergence between patches. However, because wrentits have short dispersal distances, small territories, and relatively specific habitat requirements, it is possible that wrentits could be affected by habitat fragmentation.

The landscape of southern California continues to be rapidly altered by urbanization and the resulting habitat loss and fragmentation, even though it is part of the California Floristic Province and is one of Conservation International's world biodiversity hotspots ([30], [31], www.biodiversityhotspots.org). Because it is in the Los Angeles area, SMMNRA is under intense development pressure and urbanization might increase to as much as 47% of the area by 2050, whereas only 11% was urbanized in 2000 [32]. Given the low vagility of these four focal species, it is possible that movement out of suitable habitat across a highly urbanized landscape is rare. This isolation could increase the genetic divergence between populations living in fragments and also decrease genetic variability within fragments. If urbanization is not an impenetrable barrier to movement, migration between patches by individuals could mitigate negative genetic effects [4], [33], [34]. Understanding plant and animal responses to habitat destruction and fragmentation will be important for maintenance of this important biodiversity hotspot, especially in the face of unknown consequences of global climate change.

## Results

**Figure 1:**
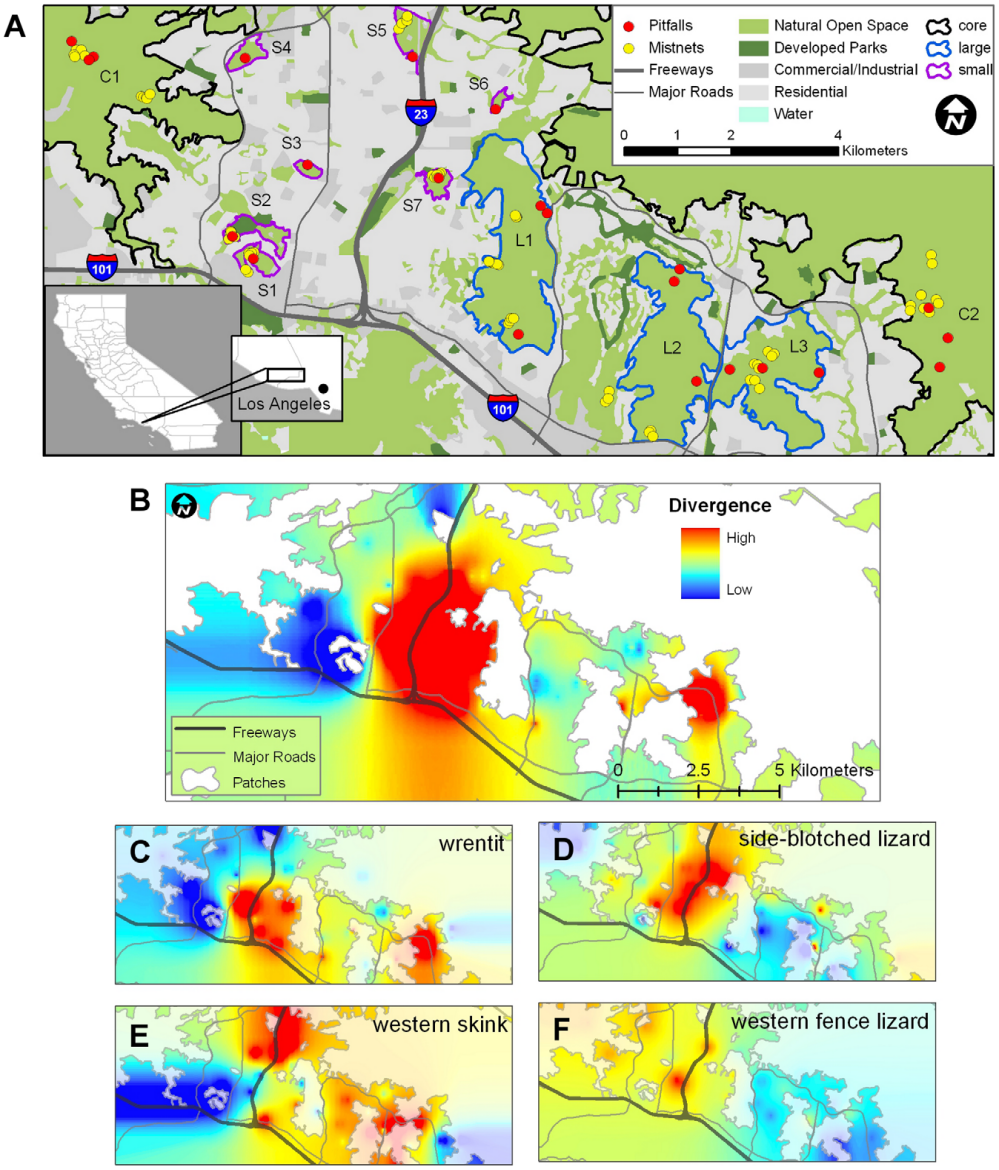
Study area and genetic divergence. A. Sampling sites (mist-net and pitfall locations), roads, and habitat patches (S = small, L = large, C = core) within the study area (Sample sizes are shown in Table 1). B. Mean genetic divergence mapped on the Simi Hills landscape for all four species, and separately for C. wrentits, D. side-blotched lizards, E. western skinks, and F. western fence lizards.

We attempted to genotype approximately 20 individuals from each species for each sample site (Fig. 1a, Table 1), although for some locations fewer than 20 were captured. Microsatellite loci in lizards did not significantly deviate from HWE, however three loci in wrentits did (Ase48, Ase64, Ase50). We didn't find an excess of homozygotes, which could indicate the presence of null alleles, at any of the three loci; so analyses were done using all loci. All microsatellite loci were in linkage equilibrium for all 4 species, except that in western skinks 2 pairs of loci were significantly linked (p = 0.05; Eufa1×Elo34, Elo34×Eufa27).

**Table 1:** Patch metrics (area, isolation, and age) and the number of samples genotyped by species.

Patch metrics					Number of samples genotyped			
Patch type	Sample site/patch	Area (ha)	Isolation (PROX)^a^	Age (years)^b^	Wrentit	Side-blotched lizard	Western fence lizard	Western skink
Small	S1	267.2	119.3	13	0	14	14	16
	S2	376.6	115.4	13	3	15	18	17
	S3	104.8	52.8	33	0	18	0	18
	S4	254.8	6404.8	23	0	17	0	0
	S5	450.2	195.9	33	5	14	18	5
	S6	78.2	747.4	13	0	17	0	20
	S7	206.5	133.1	43	8	15	16	10
Large	L1	4445.4	18428.1	28	7	0	18	28
	L2	3905.7	1598.1	23	8	22	17	29
	L3	3276.1	30121.0	18	12	18	17	18
Core	C1	25453.6	6368.9	23	11	7	15	0
	C2	121014.2	10718.8	13	15	24	14	18

a Patch isolation values (PROX) decrease with increasing isolation of patches.

b Patch age was calculated as the number of years since the patch was 100% isolated from other open natural space.

### Genetic Divergence

Pairwise F_ST_ values indicated many significant genetic differences between patches for all four species (84% of comparisons were significant for side-blotched lizards, 89% for fence lizards, 87% for skinks, and 71% for wrentits; Table S1). Average pairwise F_ST_ between patches was highest in the wrentit at 0.095 (range 0.012–0.299). Among lizards, the level of differentiation was highest for side-blotched lizards, with an average pairwise F_ST_ of 0.073 (range −0.006–0.200), and very similar for western skinks (mean F_ST_ = 0.040, range 0.003–0.104) and western fence lizards (mean F_ST_ = 0.040, range 0.003–0.095). As a baseline comparison from continuous habitat, when we computed genetic distances between the sampling arrays within large and core patches and between several other sites outside of our urban study area (but within the park, see Methods), we found lower average F_ST_ for all three lizard species (side-blotched lizards, 0.02; western fence lizard, 0.016; western skinks, 0.013), and fewer significant pairwise F_ST_ (side-blotched lizards, 12.5%; western fence lizards, 16.7%; western skinks, 30%; Table S2). For wrentits, genetic samples were also collected from two coastal canyons outside of our study area, and the F_ST_ between these two sites was non-significant (F_ST_ = 0.026). Significant genetic distances between patches could also be caused by isolation by geographic distance. We found no significant correlations between genetic distance (F_ST_) and geographic distance in any of the four species, suggesting no pattern of isolation by distance (Table 2). However, partial Mantel tests showed that genetic distances for all four species were significantly correlated with highway presence, roads presence, and time since isolation (patch age) when geographic distance was held constant (Table 2).

**Table 2:** Mantel and partial Mantel tests with genetic distance and landscape features.

Mantel Tests	Wrentit		Side-blotched lizard		Western skink		Western fence lizard	
	r	*p*	r	*p*	r	*p*	r	*p*
F_ST_ and GD^a^	−0.015	0.500	−0.011	0.509	0.178	0.162	0.042	0.408
Partial test, HWY^b^	0.430	0.001	0.259	0.027	0.442	0.007	0.255	0.049
Partial test, RDS^b^	0.425	0.031	0.314	0.015	0.495	0.012	0.399	0.016
Partial test, AGE^b^	0.458	0.009	0.393	0.033	0.466	0.045	0.760	0.002

a Mantel test correlations between genetic distance (F_ST_) and geographic distance (GD).

b Partial Mantel tests for partial correlations between the presence of Highway 23 only (HWY), the presence of major roads including Highway 23 (RDS), and the age of isolation between patches (patch age; AGE) while controlling for geographic distance.

Alleles in Space allows for visualization of genetic divergence over geographic space. We found that the largest area of genetic divergence for all four species was located in the area surrounding and including Highway 23 (Fig. 1b). There was also an area of higher divergence in the eastern part of the study area for two of the four species (wrentits, Fig. 1c; and western skinks, Fig. 1e).

**Figure 2:**
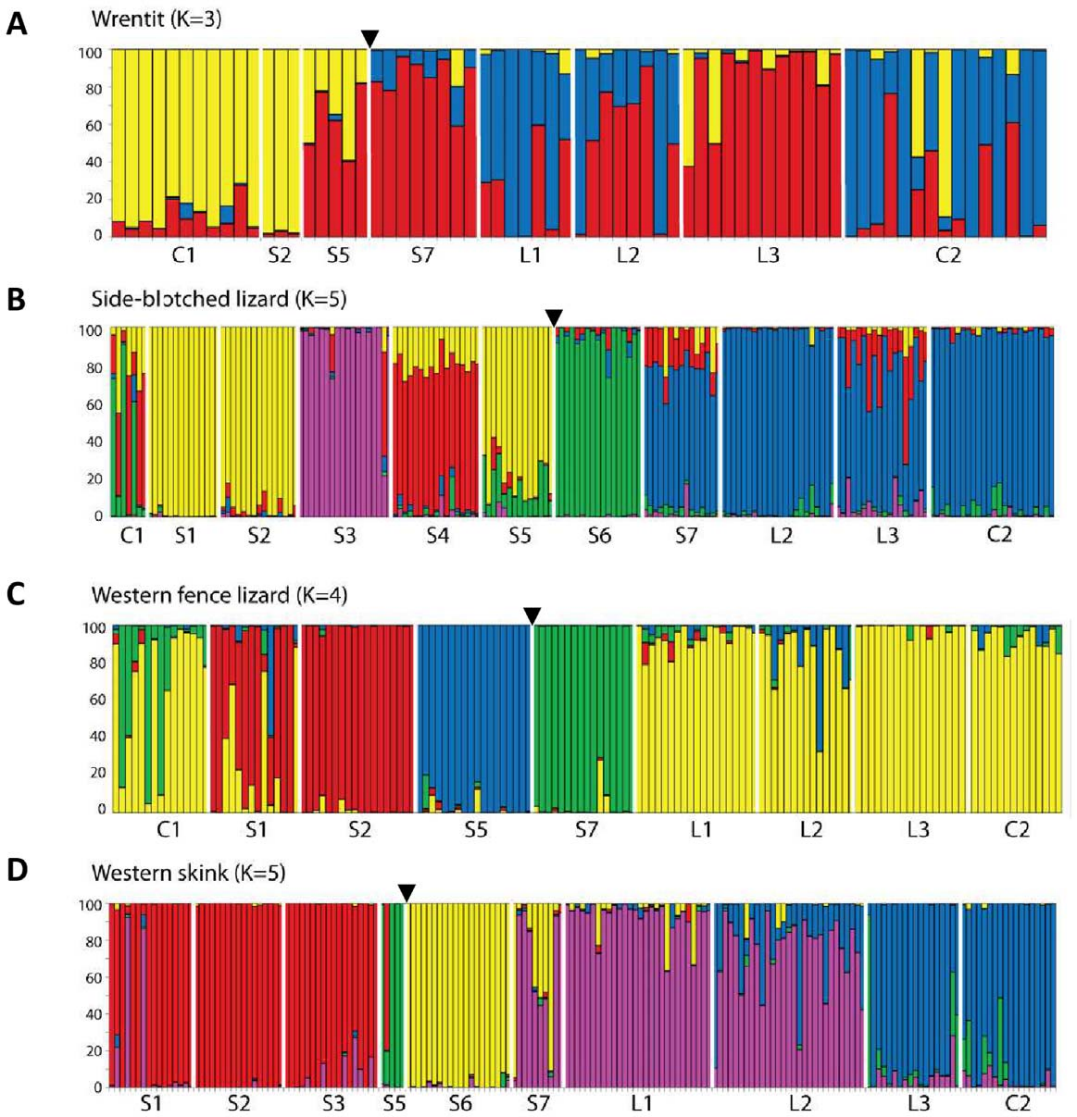
Genetic clustering analysis reveals the most likely K. Each column represents one individual and colors correspond to the percentage of assignment to each cluster. Patch names (Fig. 1a) are on the X-axis organized from west (left) to east (right). A black triangle indicates the location of the 23 freeway.

Genetic clustering analysis revealed that the most likely number of genetic groups for all four species was between three and five (Table S3, Fig. 2). For wrentits (Fig. 2a) there were three most likely clusters, with the main genetic break again located across the developed areas surrounding and including Highway 23. For side-blotched lizards (Fig. 2b) and skinks (Fig. 2d) the most likely number of clusters was five, and for western fence lizards (Fig. 2c) it was four.

### Genetic Diversity

Mean heterozygosity (H_e_) and the mean number of effective alleles (N_A_) were not significantly lower in smaller patches for any of the four species (Table S4). However, relatedness was higher in small patches for all three lizard species (side-blotched lizard difference = 0.03, *t* = 4.1, *p* = 0.003, d.f. = 6.2; fence lizard difference = 0.02, *t* = 4.5, *p* = 0.001, d.f. = 7; skink difference = 0.02, *t* = 2.25, *p* = 0.03, d.f. = 6). Rarefaction analysis indicated that the number of loci used produced consistent average relatedness results for all species and that the addition of the last locus added a 0.5% (fence lizards), 0.8% (side-blotched lizards), 1.4% (western skinks), and 0.1% (wrentits) change in relatedness estimates.

**Figure 3:**
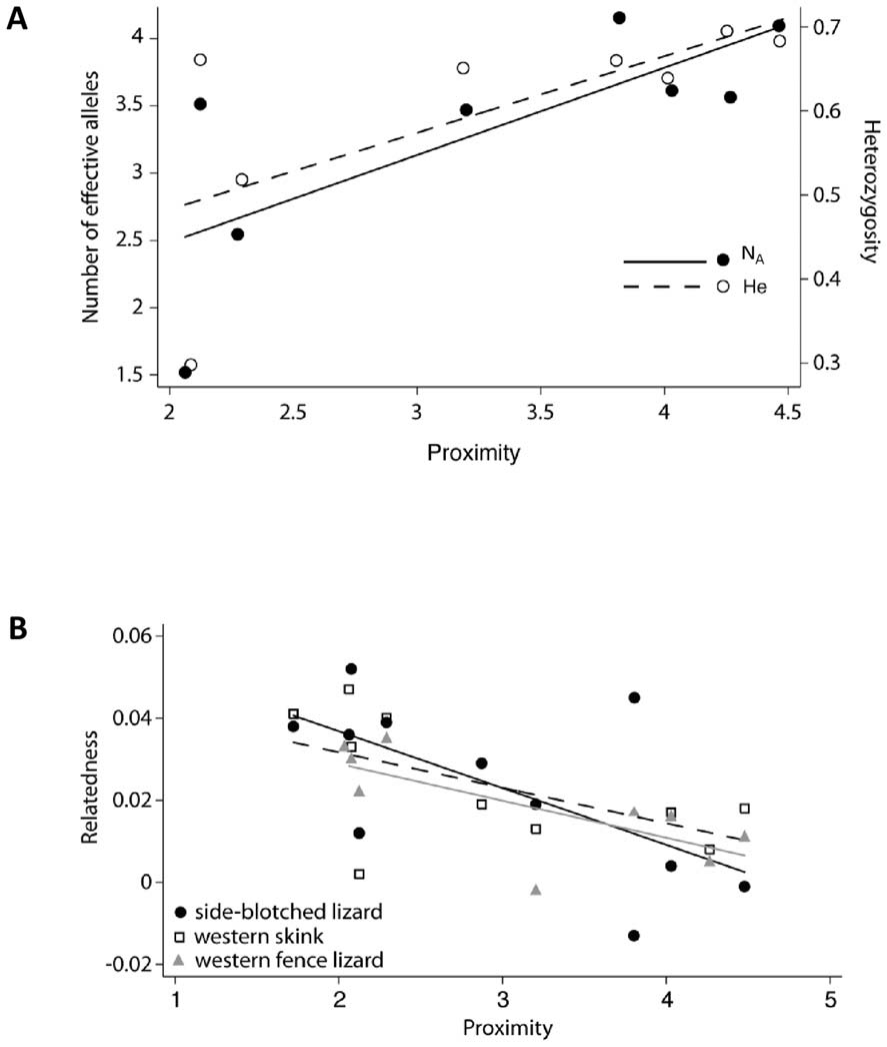
Relationship between genetic diversity (heterozygosity, number of effective alleles, and relatedness) and patch proximity^a^ (log transformed). A. wrentits (H_e_: R^2^ = 0.698, p = 0.051, d.f. = 7; N_A_: R^2^ = 0.55, p = 0.035, d.f. = 7) and B. three lizard species (R_LR_: side-blotched lizard R^2^ = 0.4, p = 0.03, d.f. = 10; western skink R^2^ = 0.33, p = 0.05, d.f. = 9; western fence lizard R^2^ = 0.52, p = 0.002, d.f. = 8). ^a^ Patch proximity is the inverse of patch isolation.

We tested the relationship between genetic diversity and the degree of isolation of each habitat patch and found that for wrentits, H_e_ was lower in more isolated patches (*R^2^* = 0.498, *p* = 0.051, d.f. = 7), as was N_A_ (*R^2^* = 0.55, *p* = 0.035, d.f. = 7; Fig. 3a). Relatedness was higher in more isolated patches for all three lizard species (side-blotched lizard *R^2^* = 0.4, *p* = 0.03, d.f. = 10; fence lizards *R^2^* = 0.52, *p* = 0.002, d.f. = 8; western skink *R^2^* = 0.33, *p* = 0.05, d.f. = 9; Fig. 3b). There were no correlations between genetic diversity and patch age for any of the four species (Table S5).

## Discussion

### Loss of genetic connectivity

Using three different methods, traditional pair-wise genetic distance analysis (F_ST_; Table S1), landscape genetic analysis (Fig. 1b), and Bayesian genetic clustering (Fig. 2), we found significant genetic differences between sample locations in all four species. Moreover, the three methods showed strikingly similar and strong genetic effects of fragmentation. All four species exhibited the largest genetic divergence over the oldest (based on building dates, see Methods) and widest expanse of urban areas surrounding and including Highway 23 (Fig. 1b).

Pairwise F_ST_ between habitat patches showed that the genetic divergence was significant, especially given the short amount of time that the habitat fragments have been isolated from each other and from core areas. For all four species, average F_ST_ values within continuous habitat were 2.5 to 3.6 times lower than in fragmented habitat, and the majority of comparisons were non-significant (Table S2). This suggests that microsatellite allele frequencies within and between habitat patches are changing on a very short time scale. Several other reptile and amphibian studies have found similar genetic divergences on similar time scales. For example, genetic divergence between fragmented populations of two gecko species in Australia was higher than divergence between samples in un-fragmented landscapes [35]. In that study, forested habitat patches were fragmented beginning around 1900 by wheat fields, which can be dry and barren during the non-growing season. In our study, however, the intervening landscape is concrete, asphalt, buildings, or urban yard landscaping, and although fragmentation began in the 1940s, many patches were only 50–75% isolated until 1980, making the isolation more recent. The long-lived tuatara (*Sphenodon punctatus*) was shown to have small yet significant genetic structuring (overall R_ST_ = 0.012) over less than 500 meters on a recently fragmented island [18]. Overall divergence was driven by one remnant forest fragment which was most isolated by island topography. Therefore, it was unclear that human activity, in this case pasture cleared for livestock grazing, was the cause of the genetic divergence. The eastern red-backed salamander (*Plethodon cinereus*), an even smaller and less mobile animal then the lizards we investigated, had pairwise F_ST_ between patches similar in value to the lizards in our study [14]. In that study, habitat fragmentation was also caused by 20^th^ Century urbanization.

For birds, few studies have shown large pairwise F_ST_ between patches on such a small scale. For example, a study of the capercaillie (*Tetrao urogallus*) in the Black Forest in Germany showed significant pairwise F_ST_ between sites, ranging from 0.007 to 0.036 [36]. In their study area, which was approximately 10 times the size of ours, suitable forest habitat was fragmented by other forest types and grassland, as opposed to by residential and commercial development. A study [37] of white-ruffed manikins (*Corapipo altera*) showed similar results to the capercaillie. There was some genetic structuring shown between remnant forest fragments, however all significant pairwise F_ST_ could be attributed to one forest fragment. In addition, pairwise F_ST_ between forest fragments ranged from 0.001 to 0.029 for manikins, whereas in our study the pairwise F_ST_ were approximately 10 times higher. Finally, a study of great tits (*Parus major*) in forest parks within the city of Barcelona found many significant pairwise F_ST_ between parks (average 0.067), but the parks actually had higher genetic diversity than the surrounding forest, and there was significant gene flow both between parks and from the parks to the forest [38]. Overall, there are few comparable studies of avian fine-scale genetic structure, particularly in urban landscapes, but wrentits in southern California appear to have the highest amount of genetic structure documented to date.

The Bayesian clustering analysis confirmed the loss of genetic connectivity for each species in our study area. Similar analyses in other bird studies have consistently shown that one genetic cluster is most likely [36], [37], [39], [40], with only the great tit study finding two clusters [38]. For the lizards, in many of the small patches most or all individuals were given close to 100% assignment to that patch (e.g. S3 for side-blotched lizards, S5 and S7 for fence lizards, and S6 for skinks; Figure 2), which suggests a remarkable amount of genetic isolation within patches over relatively short geographic and temporal scales (Table S6). The short dispersal distances for all four species suggest that gene flow even within the natural landscape may be limited (for lizards, we did find a few significant F_ST_ values between sampling sites within continuous habitat), and therefore may be extremely restricted in a fragmented landscape. In one of the few other studies using Bayesian genetic clustering analysis, red-backed salamanders (*Plethodon cinereus*) were found to have two genetic clusters on either side of a large highway running through the urbanized study area [41]. Our genetic clustering results suggest that the intense fragmentation from urbanization may be a particularly strong barrier to animal movement and gene flow for all four species.

Along with significant divergence between patches, we also found significant correlations between specific causes (roads) and measures (patch age) of fragmentation and genetic divergence in all four species (Table 2). Further, our landscape genetic results revealed that the areas surrounding and including Highway 23 in the city of Thousand Oaks, which are the oldest and most densely urbanized, consistently had the highest peaks of differentiation, again for all four species (Fig. 1b). The concordance of these results for all four species is remarkable given their differences in mobility, ecology, and taxonomy. A second area of high genetic divergence in the eastern portion of our study area, also characterized by a major road surrounded by a wide swath of residential development, was shared by two species, wrentits and skinks. Other species have also shown fine-scale genetic changes related to roads and fragmentation in this region. Coyotes and bobcats exhibited significant genetic differentiation across Highway 101, the largest highway in the study area [42]. It is unknown if the species in our study would cross such a large barrier, but with short dispersal distances and small home range sizes, those events would likely be rare. Similarly, in smaller and less mobile species, a loss of genetic connectivity and diversity was found in two Jerusalem crickets (*Stenopelmatus ‘santa monica’* and *Stenopelmatus ‘mahogani’*) across the same region [16], [43]. Genetic divergence in Jerusalem crickets was significantly associated with urban development and the presence of highways within the Simi Hills.

The significant genetic divergence and loss of genetic diversity over short geographic and temporal scales in these four vertebrates suggest that the urban matrix is relatively impenetrable for these animals. Anecdotal observations suggest that *S. occidentalis*, but not *P. skiltonianus* or *U. stansburiana*, will move through or persist in the residential areas of the urban matrix (RNF personal observation). However, reliable data on the urban movement and habitat use of these species does not exist. In fact, knowledge about use of the urban matrix by native animal species is extremely limited in general, but would be very valuable for understanding the conservation and management implications of urbanization. Urbanized areas may be dangerous places for these small vertebrates. Residential neighborhoods often introduce predators such as domestic cats, which may regularly prey on native vertebrates [44]. Of course residential areas also include roads, which lizards and birds may actively avoid, or which may be a significant source of mortality [45], [46], [47].

### Loss of genetic diversity

When the landscape is fragmented and gene flow is restricted, as we have shown for these four species, genetic diversity may be reduced in populations within smaller or more isolated habitat patches. Although we found no relationships between patch age and genetic diversity, we found significant relationships between genetic diversity measures and patch size or isolation for all four species. All three lizards had increased relatedness in smaller patches and with increasing patch isolation (Fig. 3b). Other reptile species have shown increased relatedness within habitat patches that were fragmented by agriculture [48], [49], [50]. In wrentits, although we did not find increased within-patch relatedness, we found lowered heterozygosity (H_e_) and fewer alleles (N_A_) in smaller patches (Fig. 3a). Decreased gene flow can result in decreased H_e_ and N_A_ in small patches as alleles are lost over the generations. This effect tends to be gradual and may not threaten populations in the short term, however, inbreeding within habitat patches tends to happen quickly and can lead to inbreeding depression [51]. Lizard relatedness values suggest that inbreeding is occurring within smaller and more isolated patches. The difference between taxa may be attributed to the increased effective isolation of lizards on suitable habitat patches as a result of more restricted dispersal ability compared to wrentits. Our results suggest that populations within smaller and more isolated patches may have an increased risk of harmful genetic effects and, over the long-term, even extirpation. In fact, the absence of individuals from certain study patches (e.g. skinks and fence lizards absent from S4; Table 1) suggests that populations that were presumably present at the time of patch isolation may have been extirpated.

In a relatively short time, we have documented significant genetic divergence between isolated patches and decreased genetic diversity in all four species. However, although time since isolation (patch age) was strongly correlated with genetic divergence between patches, the effects on genetic diversity in these animals were significantly related to patch size and degree of patch isolation, but not to patch age. This would suggest that the habitat is still relatively suitable in habitat fragments, resulting in relatively stable populations that are not going through bottlenecks, such that more time since isolation is not as important a factor. But patches that are smaller from the outset simply cannot support as large a population, and therefore are more subject to the deleterious effects of genetic drift, specifically the loss of genetic diversity. Patches that are more isolated may in turn be less likely to receive new dispersers, i.e. they would benefit less from the “rescue effect” that could offset reductions in genetic diversity [52]. Presumably patches that were both small and isolated would suffer the most ill effects.

### Conservation implications

The extreme urbanization within the Simi Hills area has had a significant effect on lizard and bird population genetics. Unlike some other studies of landscape level genetic changes where a species' habitat is naturally patchy, this study examined genetic responses to species living in habitat that was likely once relatively continuous [42]. While these species are still widely distributed and relatively abundant throughout the study area, genetic effects of fragmentation have been manifested in a relatively short period of 40 years or less. This may be the most profound and potentially disturbing result of our study: the vulnerability even of species that are perceived to be common and thereby likely less affected by habitat fragmentation. This may be particularly true for low-vagility organisms, and for those with more specific habitat requirements. As a chaparral and coastal sage scrub requiring species, wrentits are likely rare in developed areas and have been shown to go extinct in habitat patches as urbanization progresses [2], [53], [54].

For rarer species in the region, such as horned lizards (*Phrynosoma coronatum*) and whiptail lizards (*Aspidoscelis tigris*), whose distributions have already been reduced by urban development [55], the genetic effects of fragmentation may be even more profound. Many endangered species in southern California are declining because of habitat loss, and many of these species also have low dispersal abilities along with more specific habitat requirements (e.g. light-footed clapper rail, *Rallus longirostris obsoletus*; Belding's savannah sparrow, *Passerculus sandwichensis beldingi*; red-legged frog, *Rana draytonii*; least bell's vireo, *Vireo bellii pusillus*). It is also unknown how stressors, such as increasing local or global temperature and urbanization, might affect species in southern California. A recent study of *Sceloporus* lizards in Mexico found that 12% of local populations have gone extinct since 1975 [56]. Sites where these common lizards were extirpated were too hot for too many hours of the day, presumably due to increasing global temperatures, which caused lizards to seek refuge from the heat instead of spending time foraging. In addition, our results have implications for endangered species such as the California gnatcatcher, where lack of differentiation at certain loci (e.g. mtDNA; [57]) may not reflect important genetic differentiation detectable with other markers such as microsatellites.

## Materials and Methods

### Study Area

Southern California is characterized by a Mediterranean climate with cool, wet winters and hot, dry summers. Vegetation consisted of coastal sage scrub, chaparral, riparian habitat, and oak woodlands. Our study site is within SMMNRA, the USA's largest urban national park (154,095 acres or 623.6 km^2^; www.nps.gov/samo/parkmgmt/statistics.htm), which is located in Los Angeles and Ventura counties, California, USA (Fig. 1a). Approximately half of the land within the park boundary is privately owned, although some public acquisitions continue. Habitat patches within our study area were within 12.5 kilometers (km) of each other but were separated by roads of all sizes, housing, and commercial development (Fig. 1a). Most building started in the middle of the 20^th^ Century, and none of the habitat patches have been completely isolated for longer than 43 years (Table 1; [58]). Two major freeways (101 and 23) and many busy four-lane roads run through the study area (Fig. 1a). The peak average daily traffic in this area is approximately 180,000 cars per day for the 101 Freeway and 90,000 cars per day for Highway 23 (Caltrans, www.ca.dot.gov). Both freeways are mostly surrounded by commercial and residential development. Within the study area there are large core areas of relatively undisturbed habitat, although some low-impact human recreation does occur. Within the urban mosaic, habitat patches were surrounded by high- or low-density housing, highways and other roads, golf courses and other landscaped areas.

We collected samples from habitat fragments which we characterized as “small” (75–450 ha) or “large” (3200–4400 ha) and from larger areas of continuous habitat which we called “core” areas (Fig. 1a). There were 7 small patches (S1-S7), 3 large patches (L1-L3) and 2 core areas (C1 and C2). Patch area (m^2^) and degree of isolation (PROX) were calculated using FRAGSTATS [59]. PROX is the sum of patch area divided by the nearest edge-to-edge distance squared between all of the patches within a defined search radius and the focal patch. PROX approaches 0 if the patch has no neighbors within the search radius (a 20 km radius encompassed our entire study site) specified in FRAGSTATS, therefore patches with smaller PROX numbers are more isolated. Building dates for roads, housing developments, and commercial areas were used to calculate the ages (in years) at which patches were 100% isolated up to the time of trapping for this study (patch age; Table 1). Patches were considered 100% isolated when they were completely surrounded on all edges by either commercial buildings, housing, or roads or a combination of these. We also made a matrix of patch ages (for pair wise comparisons) by calculating the number of years that each patch was separated from each other patch.

### Field sampling

To capture lizards we used arrays of pitfall traps and drift fencing. All samples for this study were collected between October 2000 and September 2005. Each array had seven 19-liter buckets buried in the ground with the lip of the bucket flush with the ground to act as a pitfall trap [55], [60]. Buckets were arranged in a “Y” configuration and buried approximately 7.5 m apart. Between the buckets, short drift fencing (0.5 m tall) consisting of erosion cloth acted to intercept reptiles moving through the habitat and directed them towards the buckets. Shade and moisture were provided for each bucket to maximize the chance of survival for reptiles, amphibians, or small mammals that were trapped. Pitfall traps were checked daily for a week at one-month intervals [22]. Each reptile was identified to species and snout to vent length was measured in mm. Each individual was assigned a unique number, was permanently marked by toe clipping [61], [62] and a small sample from the tip of the tail was taken. Toes and tail tips were stored in 70% ethanol at 4°C or −80°C depending on storage space.

To capture birds, we used mist-nets. Trapping occurred from August 2004 to May 2006. Generally, we would open mist-nets (9–12 m long, 30 cm mesh) at sunrise and close them as the temperature increased to a potentially unsafe level in mid-morning. We targeted wrentits by playing male territorial songs with portable speakers placed at the base of the net. Once a bird was caught in the net, it was immediately removed and a U.S. Fish and Wildlife Service band was placed on its leg. We also took measurements of culmen length (mm), culmen width (mm), unflattened wing chord length (cm), tail length (cm), tarsus length (cm) and mass (g). Culmen length was taken from the anterior end of the nares to the tip of the beak using calipers. For genetic samples, we punctured the brachial vein on the wing of each bird with a small gauge needle and collected the blood that pooled there with a small capillary tube. Bleeding usually stopped after 10 seconds which yielded approximately 100 µl of blood. Blood was then placed in avian blood buffer [63].

All samples used in this study came from animals that were captured, handled, and released according to relevant national and international scientific guidelines. We used common field and handling methods that minimize stress and long-term effects of capture. We also researched methods alternative to toe-clipping of reptiles and determined that there were no less harmful yet permanent ways of marking individuals [62]. We obtained approval for our animal capture protocol from the UCLA Office of the Protection of Animals (OPRS).

We extracted genomic DNA with the Qiagen DNA mini kit (Qiagen Inc.). DNA samples were stored in TE buffer (10 mM Tris-Cl pH 8.0, 1 mM EDTA pH 8.0) at −20°C. We used six to eight microsatellite markers for each species (Table S6, J. Archie, Pers. Comm.; [64], [65], [66], [67]). We used flourescently-labeled forward microsatellite primers when available. Alternatively, we used a three-primer genotyping protocol, where the forward microsatellite primer had an M13 sequence attached to the 5′ end (5′-GTAAAACGACGGCCAG-3′) and a third primer with the complementary M13 sequence was dye-labeled [68], [69]. The forward, reverse and M13-dye primers were then used in a three-primer PCR protocol using Multiplex Mix (Qiagen Inc.) and 0.01% Bovine Serum Albumin (BSA) to generate microsatellite alleles which are flourescently labeled. Genotypes were run on an ABI 3700 sequencer and alleles were visualized using GENEMAPPER (Applied Biosystems, Inc.).

### Genetic Analysis

We used the computer program CONVERT to translate our microsatellite genotype files into the correct input format for various analysis programs [70]. We used FSTAT 2.9.3 [71] to test for deviations from Hardy-Weinberg equilibrium (HWE) within samples using 1000 permutations. We also used FSTAT to test for linkage disequilibrium (LD) between loci. P-values were adjusted for multiple tests using a sequential Bonferonni correction [72]. For HWE and LD, all samples for each species were assumed to be a single population.

#### Genetic divergence

We used the program ARLEQUIN to estimate pair-wise F_ST_ values between patches using the infinite-allele model and 1000 permutations for significance [73], [74]. We also calculated pair-wise F_ST_ between arrays within large and core patches with ARLEQUIN to show genetic divergence between sampling sites that were located within a patch of continuous habitat. For this calculation we also included some sampling sites from core areas of continuous habitat that were outside of the Simi Hills (our study area), but within SMMNRA, with an average of 4.28 km (range 1.8–6.6 km) separating these sites.

To examine patterns of sample clustering based on genetic similarity, we used the program STRUCTURE v. 2.3.1 [75]. We chose the LOCPRIOR model [76], assumed populations were not admixed and that allele frequencies were correlated between populations, and ran 100,000 MCMC chains with a 10,000 burn-in. We ran seven runs each of K = 1 to K = number of sample sites (Fig. 1a) for each species. We compiled results from our STRUCTURE runs with the program STRUCTURE HARVESTER (Dent Earl, http://taylor0.biology.ucla.edu/struct_harvest/). To determine the most likely K, we calculated the posterior probabilities of the mean of seven runs at each K (Table S3; [75]).

Isolation by distance, as revealed by a correlation between pairwise genetic and geographic (Euclidean) distances using a Mantel test, was performed using IBDWS 3.14 [77]. IBDWS uses a Reduced Major Axis (RMA) regression to estimate the slope and intercept of the isolation by distance relationship.

To test for the effect of major roads, highways, and patch age on genetic divergence, we performed partial Mantel tests [78] in IBDWS 3.14. Partial Mantel tests determined correlations of roads presence (RDS), highway presence (HWY), and patch age of isolation (AGE) on a genetic divergence matrix, while holding geographic distance constant. Tests were performed separately, one for each of these three variables, and all animals that were captured within a patch were used to calculate a patch average genetic divergence (F_ST_; as calculated in ARLEQUIN, see above). The presence of major roads and the presence of Highway 23 were used separately in the analysis because the highway in our study area is larger and has more traffic than other roads. Also, several habitat fragments are only separated by major roads. Age of isolation was chosen because this measure incorporates not only when roads and freeways were built, but also when residential and commercial developments were erected.

We mapped genetic distance on the landscape using Alleles in Space (AIS) and the landscape shape interpolation [79]. We used a Delaunay triangulation-based connectivity network to identify midpoints between our sample sites, then the raw genetic distance (D_ij_) at each midpoint was calculated [79]. This genetic distance measure is similar to Nei's standard genetic distance (D_s_; [80]), where D_ij_ is 0 if individuals are completely genetically identical, and D_ij_ is 1 if individuals are completely genetically dissimilar. We did not calculate the residual genetic distance, because we did not find a significant isolation by distance effect in the Simi Hills samples for any species (see Results). By this method, a landscape of genetic distances between sampling sites are expressed as “surface heights” and are displayed as a 3-dimensional graph. To better visualize the AIS height output, we imported the output file into ArcGIS 9.3 (ESRI Corporation, Redlands, CA) and created a 2-dimensional color hot-spot map overlaid on the geographic study area. Colors correspond to “heights” of genetic distance between points (e. g. Fig. 1b).

#### Genetic diversity

We used the program GENALEX [81] to calculate the genetic diversity indices of within-patch expected heterozygosity (H_e_), observed heterozygosity (H_o_), number of effective alleles (N_A_), and relatedness (R_LR_) [82]. We used the Lynch & Ritland (1999) estimator of relatedness because it has been shown to perform well in simulations for a wide range of marker data and population structure [83]. We performed a rarefaction analysis using the web-based program RERAT [84] which uses multiple simulations to determine the change in relatedness values as additional microsatellite loci are added. In RERAT, we performed 100 simulations and used the Lynch and Ritland (1999) relatedness analysis for each of the four species. For lizards, cores and large patches had three pitfall trap arrays while small patches had one (Fig. 1a). To reduce bias because of array clustering, we calculated pair wise relatedness of all individuals caught in the same array, and then used the mean of those within-array measures to calculate within patch relatedness.

We used the program STATA 9 (StataCorp, College Station, TX) to transform variables until they approached normal distributions and then to examine the relationship between the indices of genetic diversity and the size, degree of isolation, and age of the habitat patches. We used unpaired t-tests (with unequal variance when necessary) and Bonferroni corrections to compare genetic diversity measures between small and large/core habitat patches. Degrees of freedom for t-tests were calculated using the Satterthwaite (1946) method [85]. We lumped large patches and core areas for this analysis because, for these small species, population size is likely equivalently large in the large patches and the core areas, and because the numbers of sites were relatively small for core areas (n = 2) and large patches (n = 3). To test for a relationship between patch isolation and genetic diversity, we used linear regression to examine the relationship of the genetic diversity indices with the size, pair wise age of isolation, and proximity (PROX) of the habitat patches, where the degree of isolation of a patch is the inverse of proximity. Spearman's rank correlations were used to test for significant associations between patch age and genetic diversity.

## Supporting Information

Table S1 F_ST_ between sample sites for 4 species. Significant pairwise F_ST_ values are in bold (see Fig. 1a for sample site locations).
(0.22 MB DOC)

Table S2 Pairwise F_ST_ and the number of significant comparisons between patches in and continuous habitat.
(0.05 MB DOC)

Table S3 Estimated posterior probabilities for K. Most likely number of genetic clusters (K) identified with the program Structure is shown in bold.
(0.07 MB DOC)

Table S4 Mean genetic diversity measurements within patches (number of effective alleles, N_A_; relatedness, R_LR_; heterozygosity, H_e_).
(0.12 MB DOC)

Table S5 Spearman's Rho correlation coefficients. The number of individuals genotyped (N), the number of alleles (A), expected (H_e_) and observed (H_o_) heterozygosity.
(0.04 MB DOC)

Table S6 Microsatellite primers used for each species. The number of individuals genotyped (N), the number of alleles (A), expected (H_e_) and observed (H_o_) heterozygosity.
(0.14 MB DOC)
