# Supplementary material for: A Rapid, Strong, and Convergent Genetic Response to Urban Habitat Fragmentation in Four Divergent and Widespread Vertebrates
Source: PLoS One. 2010 Sep 16;5(9):e12767. doi: 10.1371/journal.pone.0012767 (PMC2940822; doi:10.1371/journal.pone.0012767)
Supplement: Table S1 — FST between sample sites for 4 species. Significant pairwise FST values are in bold (see Fig. 1a for sample site locations). (0.22 MB DOC) [file pone.0012767.s001.doc]

1. wrentit

| site | S2 | S5 | S7 | L1 | L2 | L3 | C1 |
| --- | --- | --- | --- | --- | --- | --- | --- |
| S5 | **0.274** |  |  |  |  |  |  |
| S7 | **0.299** | **0.075** |  |  |  |  |  |
| L1 | **0.234** | **0.136** | **0.067** |  |  |  |  |
| L2 | **0.234** | **0.082** | 0.016 | 0.035 |  |  |  |
| L3 | **0.165** | 0.047 | 0.032 | **0.048** | 0.012 |  |  |
| C1 | **0.140** | **0.106** | **0.095** | **0.058** | 0.034 | **0.038** |  |
| C2 | **0.213** | **0.069** | **0.051** | 0.026 | 0.012 | **0.029** | **0.059** |

1. side-blotched lizard

| site | S1 | S2 | S3 | S4 | S5 | S6 | S7 | L2 | L3 | C1 |
| --- | --- | --- | --- | --- | --- | --- | --- | --- | --- | --- |
| S2 | **0.027** |  |  |  |  |  |  |  |  |  |
| S3 | **0.080** | **0.062** |  |  |  |  |  |  |  |  |
| S4 | **0.045** | **0.050** | **0.063** |  |  |  |  |  |  |  |
| S5 | 0.012 | **0.045** | **0.051** | **0.034** |  |  |  |  |  |  |
| S6 | **0.045** | **0.039** | **0.035** | **0.059** | **0.028** |  |  |  |  |  |
| S7 | **0.065** | **0.046** | **0.040** | **0.042** | **0.039** | **0.031** |  |  |  |  |
| L2 | **0.078** | **0.037** | **0.028** | **0.035** | **0.051** | **0.034** | **0.015** |  |  |  |
| L3 | **0.079** | **0.057** | 0.017 | **0.042** | **0.036** | **0.029** | **0.034** | **0.020** |  |  |
| C1 | **0.046** | **0.051** | **0.091** | **0.062** | **0.061** | 0.025 | **0.061** | **0.078** | 0.038 |  |
| C2 | **0.054** | **0.040** | 0.011 | **0.039** | **0.020** | 0.011 | 0.009 | -0.006 | 0.010 | **0.061** |

1. western skink

| site | S1 | S2 | S3 | S5 | S6 | S7 | L1 | L2 | L3 |
| --- | --- | --- | --- | --- | --- | --- | --- | --- | --- |
| S2 | **0.026** |  |  |  |  |  |  |  |  |
| S3 | **0.052** | **0.064** |  |  |  |  |  |  |  |
| S5 | **0.062** | **0.104** | **0.095** |  |  |  |  |  |  |
| S6 | **0.021** | **0.042** | **0.066** | **0.059** |  |  |  |  |  |
| S7 | 0.029 | **0.041** | **0.045** | **0.065** | 0.010 |  |  |  |  |
| L1 | **0.027** | **0.057** | **0.049** | **0.059** | **0.017** | 0.011 |  |  |  |
| L2 | **0.030** | **0.054** | **0.054** | **0.054** | **0.020** | 0.018 | 0.006 |  |  |
| L3 | **0.033** | **0.049** | **0.073** | **0.051** | **0.028** | **0.023** | **0.023** | **0.016** |  |
| C2 | **0.018** | **0.051** | **0.079** | **0.062** | **0.029** | 0.017 | **0.027** | **0.023** | **0.018** |

1. western fence lizard

| site | S1 | S2 | S5 | S7 | L1 | L2 | L3 | C1 |
| --- | --- | --- | --- | --- | --- | --- | --- | --- |
| S2 | **0.036** |  |  |  |  |  |  |  |
| S5 | **0.046** | **0.050** |  |  |  |  |  |  |
| S7 | **0.051** | **0.063** | **0.055** |  |  |  |  |  |
| L1 | **0.023** | **0.033** | **0.031** | **0.028** |  |  |  |  |
| L2 | **0.039** | **0.036** | **0.016** | **0.033** | 0.008 |  |  |  |
| L3 | **0.033** | **0.038** | **0.046** | **0.057** | 0.006 | 0.014 |  |  |
| C1 | **0.052** | **0.033** | **0.042** | **0.044** | **0.017** | **0.016** | **0.022** |  |
| C2 | **0.050** | **0.051** | **0.023** | **0.035** | **0.016** | 0.007 | **0.019** | **0.021** |
