# Supplementary material for: A Rapid, Strong, and Convergent Genetic Response to Urban Habitat Fragmentation in Four Divergent and Widespread Vertebrates
Source: PLoS One. 2010 Sep 16;5(9):e12767. doi: 10.1371/journal.pone.0012767 (PMC2940822; doi:10.1371/journal.pone.0012767)
Supplement: Table S2 — Pairwise FST and the number of significant comparisons between patches in and continuous habitat. (0.05 MB DOC) [file pone.0012767.s002.doc]

|  | **side-blotched lizard** | **western fence lizard** | **western skink** | **wrentit** |
| --- | --- | --- | --- | --- |
| **F_ST_** |  |  |  |  |
| Between Patches | 0.073 | 0.04 | 0.04 | 0.095 |
| Continuous habitat | 0.021 | 0.012 | 0.016 | 0.026 |
| **No. of significant F_ST_/No. of comparisons** |  |  |  |  |
| Between Patches | 46/55 (83.6%) | 32/26 (88.9%) | 39/45 (86.7%) | 20/28 (71.4%) |
| Continuous habitat | 1/8 (12.5%) | 2/12 (16.7%) | 3/10 (30%) | 0/1 (0%) |
