# Supplementary material for: A Rapid, Strong, and Convergent Genetic Response to Urban Habitat Fragmentation in Four Divergent and Widespread Vertebrates
Source: PLoS One. 2010 Sep 16;5(9):e12767. doi: 10.1371/journal.pone.0012767 (PMC2940822; doi:10.1371/journal.pone.0012767)
Supplement: Table S3 — Estimated posterior probabilities for K. Most likely number of genetic clusters (K) identified with the program Structure is shown in bold. (0.07 MB DOC) [file pone.0012767.s003.doc]

| K | wrentit | side-blotched lizard | western fence lizard | western skink |
| --- | --- | --- | --- | --- |
| 1 | 4.62E-16 | 1.16E-37 | 5.41E-61 | 1.7E-105 |
| 2 | 0.0003 | 9.78E-11 | 5.34E-41 | 2.85E-26 |
| 3 | **1** | 6.59E-13 | 8.1E-20 | 7.05E-17 |
| 4 | 4.64E-06 | 0.047426 | **1** | 0.001 |
| 5 | 3.69E-06 | **0.952574** | 3.44E-14 | **0.850249** |
| 6 | 8.32E-11 | 9.78E-11 | 5.08E-10 | 1.33E-06 |
| 7 | 6.81E-23 | 2.42E-13 | 3.19E-15 | 0.148789 |
| 8 | 5.04E-38 | 2.33E-36 | 4.76E-50 | 1.8E-14 |
| 9 |  | 1.39E-31 | 1.14E-80 | 8.11E-10 |
| 10 |  |  |  | 3.46E-07 |
