# Supplementary material for: A Rapid, Strong, and Convergent Genetic Response to Urban Habitat Fragmentation in Four Divergent and Widespread Vertebrates
Source: PLoS One. 2010 Sep 16;5(9):e12767. doi: 10.1371/journal.pone.0012767 (PMC2940822; doi:10.1371/journal.pone.0012767)
Supplement: Table S4 — Mean genetic diversity measurements within patches (number of effective alleles, NA; relatedness, RLR; heterozygosity, He). (0.12 MB DOC) [file pone.0012767.s004.doc]

| **Patch** | **wrentit** | | | **side-blotched lizard** | | | **western fence lizard** | | | **western skink** | | |
| --- | --- | --- | --- | --- | --- | --- | --- | --- | --- | --- | --- | --- |
|  | N_A_ | R_LR_ | H_e_ | N_A_ | R_LR_ | H_e_ | N_A_ | R_LR_ | H_e_ | N_A_ | R_LR_ | H_e_ |
| **S1** | - | - | - | 2.517 | 0.052 | 0.496 | 5.006 | 0.03 | 0.714 | 6.798 | 0.033 | 0.846 |
| **S2** | 1.510 | 0.309 | 0.286 | 2.583 | 0.036 | 0.544 | 4.456 | 0.033 | 0.698 | 6.352 | 0.047 | 0.805 |
| **S3** | - | - | - | 2.913 | 0.038 | 0.553 | - | - | - | 6.608 | 0.041 | 0.811 |
| **S4** | - | - | - | 2.492 | 0.045 | 0.498 | - | - | - | - | - | - |
| **S5** | 2.545 | 0.065 | 0.514 | 2.793 | 0.039 | 0.542 | 5.221 | 0.035 | 0.711 | 4.961 | 0.04 | 0.79 |
| **S6** | - | - | - | 3.07 | 0.029 | 0.568 | - | - | - | 9.12 | 0.019 | 0.877 |
| **S7** | 3.545 | 0.019 | 0.666 | 2.95 | 0.012 | 0.558 | 5.160 | 0.022 | 0.745 | 7.996 | 0.002 | 0.857 |
| **L1** | 3.597 | 0.045 | 0.695 | - | - | - | 4.626 | 0.005 | 0.713 | 10.24 | 0.008 | 0.887 |
| **L2** | 3.465 | 0.014 | 0.651 | 2.492 | 0.019 | 0.491 | 4.5 | -0.002 | 0.689 | 7.227 | 0.013 | 0.846 |
| **L3** | 4.097 | 0.027 | 0.683 | 2.682 | -0.001 | 0.498 | 4.415 | 0.011 | 0.679 | 7.096 | 0.018 | 0.847 |
| **C1** | 4.144 | 0.003 | 0.664 | 2.05 | -0.013 | 0.314 | 4.748 | 0.017 | 0.694 | - | - | - |
| **C2** | 3.646 | 0.009 | 0.639 | 2.924 | 0.004 | 0.558 | 4.381 | 0.016 | 0.68 | 7.347 | 0.017 | 0.824 |
