# Supplementary material for: A Rapid, Strong, and Convergent Genetic Response to Urban Habitat Fragmentation in Four Divergent and Widespread Vertebrates
Source: PLoS One. 2010 Sep 16;5(9):e12767. doi: 10.1371/journal.pone.0012767 (PMC2940822; doi:10.1371/journal.pone.0012767)
Supplement: Table S5 — Spearman's Rho correlation coefficients. The number of individuals genotyped (N), the number of alleles (A), expected (He) and observed (Ho) heterozygosity. (0.04 MB DOC) [file pone.0012767.s005.doc]

|  | **side-blotched lizard** | **western skink** | **western fence lizard** | **wrentit** |
| --- | --- | --- | --- | --- |
| **R_LR_** | 0.0518 | -0.389 | -0.068 | 0.072 |
| **H_e_** | -0.073 | -0.088 | -0.812 | 0.374 |
| **N_e_** | -0.049 | -0.119 | -0.845 | -0.121 |
