# Supplementary material for: A Rapid, Strong, and Convergent Genetic Response to Urban Habitat Fragmentation in Four Divergent and Widespread Vertebrates
Source: PLoS One. 2010 Sep 16;5(9):e12767. doi: 10.1371/journal.pone.0012767 (PMC2940822; doi:10.1371/journal.pone.0012767)
Supplement: Table S6 — Microsatellite primers used for each species. The number of individuals genotyped (N), the number of alleles (A), expected (He) and observed (Ho) heterozygosity. (0.14 MB DOC) [file pone.0012767.s006.doc]

| **side-blotched lizard^1^** | | | | | **western skink^2^** | | | | | **western fence lizard^3^** | | | | | **wrentit^4^** | | | | |
| --- | --- | --- | --- | --- | --- | --- | --- | --- | --- | --- | --- | --- | --- | --- | --- | --- | --- | --- | --- |
| **Locus** | **N** | **A** | **H_e_** | **Ho** | **Locus** | **N** | **A** | **H_e_** | **Ho** | **Locus** | **N** | **A** | **H_e_** | **Ho** | **Locus** | **N** | **A** | **H_e_** | **Ho** |
| SMCL | 164 | 12 | 0.731 | 0.63 | Elo3 | 166 | 18 | 0.815 | 0.638 | WFL7 | 146 | 13 | 0.795 | 0.74 | Ase18 | 68 | 5 | 0.737 | 0.794 |
| 10000M | 179 | 2 | 0.075 | 0.078 | Eufa24 | 167 | 40 | 0.962 | 0.88 | WFL15 | 141 | 15 | 0.905 | 0.80 | Ase48 | 64 | 29 | 0.940 | 0.344 |
| MCC | 179 | 6 | 0.175 | 0.162 | Eufa1 | 162 | 36 | 0.962 | 0.852 | WFL13 | 144 | 19 | 0.909 | 0.875 | Ase64 | 69 | 8 | 0.788 | 0.594 |
| BRTT | 181 | 9 | 0.749 | 0.696 | Elo34 | 171 | 30 | 0.949 | 0.696 | WFL14 | 137 | 4 | 0.078 | 0.08 | Ase52 | 69 | 3 | 0.413 | 0.391 |
| IGS | 181 | 10 | 0.777 | 0.724 | Eufa27 | 174 | 22 | 0.943 | 0.695 | WFL8 | 144 | 14 | 0.868 | 0.813 | Ase50 | 69 | 7 | 0.702 | 0.551 |
| NGFF | 178 | 6 | 0.687 | 0.618 | Eufa7 | 134 | 26 | 0.946 | 0.649 | WFL9 | 145 | 18 | 0.926 | 0.78 | Ase55 | 69 | 4 | 0.565 | 0.638 |
| PLKN | 177 | 14 | 0.752 | 0.734 |  |  |  |  |  | WFL11 | 134 | 11 | 0.840 | 0.59 | Ase29 | 69 | 17 | 0.890 | 0.87 |
| SPHILL | 174 | 15 | 0.686 | 0.598 |  |  |  |  |  | WFL12 | 138 | 19 | 0.862 | 0.789 |  |  |  |  |  |

^1^ Zamudio K, Sinervo B, 2000. Polygyny, mate-guarding, and posthumous fertilization as alternative male mating strategies. Proceedings of the National Acadamy of Sciences 97:14427-14432.

^2^ Coughlan JP, Kitson L, Dillane E, Davenport J, Cross TF, 2004. Characterization of six microsatellite loci in the Bermuda skink (*Eumeces longirostris*). Molecular Ecology Notes 4:678-679, and Howes BJ, Lee-Yaw A, Robertson AV, Bogart JP, Lougheed SC, 2004. Microsatellite loci from the five-lined skink (*Eumeces fasciatus*). Molecular Ecology Notes 4:237-238.

^3^ J. Archie pers. comm

^4^ Richardson DS, Jury FL, Dawson DA, Salgueiro P, Komdeur J, Burke T, 2000. Fifty Seychelles warbler (*Acrocephalus sechellensis*) microsatellite loci polymorphic in Sylviidae species and their cross-species amplification in other passerine birds. Molecular Ecology 9:2226-2231.
